# Chemical Compositions and Antioxidant Activities of Essential Oils, and Their Combinations, Obtained from Flavedo By-Product of Seven Cultivars of Sicilian *Citrus aurantium* L.

**DOI:** 10.3390/molecules27051580

**Published:** 2022-02-27

**Authors:** Natale Badalamenti, Maurizio Bruno, Rosario Schicchi, Anna Geraci, Mariarosaria Leporini, Luigia Gervasi, Rosa Tundis, Monica Rosa Loizzo

**Affiliations:** 1 Department of Biological, Chemical and Pharmaceutical Sciences and Technologies (STEBICEF), University of Palermo, Viale delle Scienze, 90128 Palermo, Italy; natale.badalamenti@unipa.it (N.B.); maurizio.bruno@unipa.it (M.B.); anna.geraci@unipa.it (A.G.); 2 Centro Interdipartimentale di Ricerca “Riutilizzo Bio-Based Degli Scarti da Matrici Agroalimentari” (RIVIVE), Università di Palermo, 90128 Palermo, Italy; 3 Department of Agricultural, Food and Forest Sciences (SAAF), University of Palermo, Viale delle Scienze, Building 5, 90128 Palermo, Italy; 4 Department of Pharmacy, Health and Nutritional Sciences, University of Calabria, 87036 Arcavacata di Rende, Italy; marilepo87@gmail.com (M.L.); luigiagervasi@gmail.com (L.G.); rosa.tundis@unical.it (R.T.)

**Keywords:** *Citrus aurantium*, GC-MS analysis, essential oil combinations, d-limonene, PCA analysis, global antioxidant score

## Abstract

In this work, seven *Citrus aurantium* essential oils (EOs) derived from flavedo of cultivars ‘Canaliculata’, ‘Consolei’, ‘Crispifolia’, ‘Fasciata’, ‘Foetifera’, ‘Listata’, and ‘Bizzaria’ were investigated. EOs were also combined in 1:1 (*v*/*v*) ratio to identify possible synergism or antagonism of actions. GC-MS analysis was done to investigate Eos’ phytochemical profiles. The antioxidant activity was studied by using a multi-target approach based on FRAP, DPPH, ABTS, and β-carotene bleaching tests. A great difference was observed in EOs’ phytochemical profiles. d-limonene (33.35–89.17%) was the main monoterpene hydrocarbon, and α-Pinene, β-myrcene, and β-linalool were identified in almost all samples. Among EOs, only C3 showed high quantitative and qualitative variability in its chemical composition. The chemical diversity of EOs was also demonstrated by PCA and HCA statistical analysis. Samples C2, C4, C5, C6, and C7 were statistically similar to each other, while C1 and C3 were characterized as having a different amount of other compounds and oxygenated monoterpenes, respectively, with respect to the other EOs mentioned. The global antioxidant score (GAS) revealed that among the tested EOs, *C. aurantium* ‘Fasciata’ EO had the highest antioxidant potential, with a GAS value of −0.47, whereas among combinations, the EO obtained by mixing ‘Canaliculata’ + ‘Bizzaria’ was the most active. Comparison by theoretical and real data on inhibitory concentration (IC_50_) and FRAP values did not reveal any significant effect of synergism or antagonism of actions to be valid in all biological applied tests. These findings, considered together, represent an important starting point to understand which compounds are responsible for the activities and their future possible industrial application.

## 1. Introduction

*Citrus aurantium* L., known as bitter or sour orange, belongs to the Rutaceae family (order Sapindales), and its genus is native to a wide area covering Asia (from India to northern China) and Oceania (Queensland, Australia) [1]. Its origin can probably be placed between southern China, northern Burma, and north-east India [2]. It is marketed and planted in tropical and temperate areas such as France, Spain, Italy, and North Africa [3]. *C. aurantium* originates as a natural hybrid between a pure male mandarin (*C. reticulata* Blanco) and pummelo (*C. maxima* Burm. F.) Merr. [1,4]. Italy and Spain are responsible for 80% of European *Citrus* production [5]. In Sicily, an average of 34% of *Citrus* fruits, predominantly orange, are processed into juices, providing about half of its weight as waste, which reaches 24.3 million tons per year [6,7,8].

The wastes deriving from *Citrus* processing (peels and seeds in primis) are an important part, and various papers have shown that by recycling and using these parts, bioactive compounds such as flavonoids, limonoids, terpenoids, and minerals can be obtained [9,10,11].

The flavedo of *Citrus* fruits is largely used to produce essential oils (EOs), which are exploited in both the food and cosmetic fields. Overall, the chemical composition and concentration of constituents of these hydro-distillates is varied and is influenced by climate, irrigation, maturation, and soil composition [12,13,14,15,16,17].

The predominant compound is almost always d-limonene, followed by other monoterpenes such as linalool, β-myrcene, and α-terpineol. In fact, a study carried out on the peel essential oil of a Croatian accession of *C. aurantium*, obtained by hydro-distillation and cold press method, identified twenty-two components, with limonene as a dominant compound in both hydro-distillated and cold press essential oil [18]. On the other hand, essential oil obtained from the flowers of *C. aurantium* was characterized by a minor amount of limonene and by moderate presence of *E*-nerolidol, α-terpineol, and α-terpinyl acetate [19], whereas the essential oils from the leaves were shown to be particularly rich in linalool, linalyl acetate, and α-terpineol [20].

**Figure 1:**
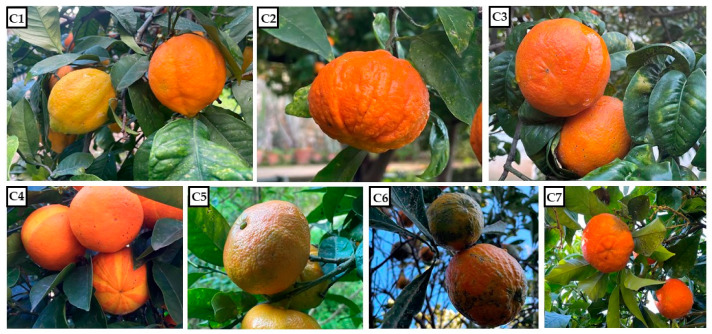
The seven *C. aurantium* cultivars grown in the Palermo Botanical Garden: *C*. *aurantium* ‘Canaliculata’ (C1), *C. aurantium* ‘Consolei’ (C2), *C*. *aurantium* ‘Crispifolia’ Risso & Poit. (C3), *C*. *aurantium* ‘Fasciata’ Risso & Poit. (C4), *C*. *aurantium* ‘Foetifera’ (C5), *C*. *aurantium* ‘Listata’ (C6), and *C. aurantium* ‘Bizzaria’ (C7).

Numerous in vitro and in vivo studies have investigated the *C. aurantium* fruits, seeds, leaves, flowers, and essential oils’ biological properties including antimicrobial, antioxidant, cytotoxic, anxiolytic, antidiabetic, anti-obesity, and anti-inflammatory activities [8,18,19,20,21,22]. Due to the abundance of health-giving secondary metabolites, *C. aurantium* is used for the treatment of several ailments, such as anxiety, cancers, gastrointestinal disorders, and obesity [23,24,25,26]. Moreover, *Citrus* EOs, due their antioxidant potential, are also used to protect foods from rancidity and/or deterioration of the nutritional quality, loss of color and flavor, alteration of texture, and auto-oxidation process. It is estimated that half of the world’s fruit and vegetable crops are lost due to post-harvest oxidative deteriorative reactions [27]. In this context, the chemical composition of EOs from different Sicilian cultivars, including *C. aurantium* ‘Canaliculata’ (C1), ‘Consolei’ (C2), ‘Crispifolia’ Risso & Poit. (C3), ‘Fasciata’ Risso & Poit. (C4), ‘Foetifera’ (C5), ‘Listata’ (C6), and ‘Bizzaria’ (C7) (Figure 1), and their combinations (1:1 *v*/*v*) were investigated by GC-MS analysis. The phytochemical diversity of the cultivars was demonstrated by HCA and PCA statistical analysis. Moreover, EOs and their combinations have been assessed for their potential antioxidant activities using different in vitro assays (FRAP, DPPH, ABTS, and β-carotene bleaching test).

## 2. Results and Discussions

### 2.1. Composition of the Essential Oils and Their Combination

Hydro-distillation of flavedo gave yellow-orange oils. Overall, twenty compounds were identified and listed in Table 1 according to their retention indices on a DB-5MS non-polar column and classified into five classes: monoterpene hydrocarbons, oxygenated monoterpenes, sesquiterpene hydrocarbons, oxygenated sesquiterpenes, and other compounds. Analyzing results, a possible division of the cultivars into two classes emerged: a cluster consisting of *C. aurantium* C1, C2, C4, C5, C6, and C7 samples, and a separate cultivar C3. The macro-group are essentially characterized by monoterpene hydrocarbons, with the majority compound d-limonene present in a variable percentage between 76.00 and 89.17%.

Among the other monoterpenes belonging to this class, and present in all the cultivars, α-pinene (1.49–3.01%) and β-myrcene (1.00–7.52%) were found. On the other hand, β-linalool (1.03–2.67%) is the only oxygenated monoterpene present unanimously in all these cultivars, and furthermore, these samples are characterized by the absence of sesquiterpenes, both hydrocarbon and oxygenated. Finally, only the C1 and C4 samples showed detectable quantities of the two aldehydes *n*-octanal (1.35–3.77%) and *n*-decanal (2.07%). The C3 sample differs enormously from the remaining samples analyzed. In the latter sample, d-limonene was present in high quantities, but much lower when compared with all the other EOs. The majority class is that of oxygenated monoterpenes (44.79%), enriched by the presence of β-linalool (7.69%), α-terpineol (7.06%), bergamol (6.77%), and geranyl acetate (10.12%). It is the only oil that showed the presence of hydrocarbon and oxygenated sesquiterpenes, while the alcohol *n*-octanol (1.59%) and the ester n-decyl acetate (1.44%) emerged in the class of other compounds.

Analyzing data reported in the literature [18,22,23,28], d-limonene remains in all cases the major compound (87.00–95.57%) extractable by hydro-distillation from the flavedo of *C. aurantium*. Furthermore, all EOs are characterized by a good quantity of α-pinene, β-myrcene, and β-linalool. EOs investigated by Farahmandfar et al. [29] were among the few EOs showing the presence of sesquiterpenes such as caryophyllene, germacrene D, and trans-nerolidol, as well as alcohol and aldehydes such as *n*-octanol, *n*-octanal, and *n*-decanal, respectively. In this case as well, however, the majority compound was the monoterpene d-limonene. Most likely, an incomplete maturation in our specimen of *C. aurantium* C3 caused this clear quantitative difference of limonene compared to all the other cultivars studied. Previously, Tundis et al. [30] reported the phytochemical profile of *C. aurantium* EO from Calabria and found a similar pattern as that found in our samples. In fact, flavedo oil contained limonene, although to a lesser extent (65.8%), followed by β-myrcene, α-pinene, linalool, and linalyl acetate. On the contrary, Abderrezak et al. [31] identified 32 compounds representing 96% of total oil content in *C. aurantium* flavedo EO from Algeria. This oil is dominated by linalool (12.0%), *trans*-carveol (11.9%), cis-linalool oxide (8.1%), and carvone (5.8%). These qualitative and quantitative differences in oil compositions depended on various factors such as genotype, season, maturational stage, and pedoclimatic factors that occur in the location of the plant’s origins [28].

Table S1 shows the composition of the combinations obtained by mixing equivolumetric quantities of the seven *C. aurantium* cultivars EOs. The different samples, from C1C2 to C6C7, were obtained by mixing two oils in equal parts; only the sample ‘mix’ (C1 + C2+ C3 + C4 + C5 + C6 + C7) was obtained for equal miscibility of the EOs extracted from the seven cultivars. The GC-MS analysis confirmed for almost all the samples, except for C1C3, C2C3, C3C4, C3C5, C3C6, and C3C7, the massive presence of compounds belonging to the class of monoterpene hydrocarbons, with a high percentage of d-limonene. Sesquiterpenes, both oxygenated and hydrocarbon, were not detected.

All these combinations were also characterized by varying amounts of oxygenated monoterpenes, a group that was exceeded by β-linalool first and by α-terpineol afterwards. Only the samples C2C5, C2C6, C2C7, C5C6, C5C7, and C6C7 were devoid of organic compounds belonging to the O class.

All the other samples, influenced by the presence of EO coming from *C. aurantium* ‘Crispifolia’ (C3), showed a lower quantity of monoterpene hydrocarbons and an enrichment of oxygenated monoterpenes, including volatile compounds such as β-linalool, α-terpineol, bergamol, and geranyl acetate. Furthermore, the peculiarity of these mixtures lies in the presence of sesquiterpenes, both oxygenated and hydrocarbon structures, with a percentage varying between 2.13 and 2.33%.

### 2.2. PCA and HCA Analyses of the Essential Oils and Their Combinations

The statistical analyses were carried out according to the loading plot obtained by principal component analysis (PCA) for monoterpene hydrocarbons (MH), oxygenated monoterpenes (OM), sesquiterpene hydrocarbons (SH), oxygenated sesquiterpenes (OS), and other compounds (O).

**Figure 2:**
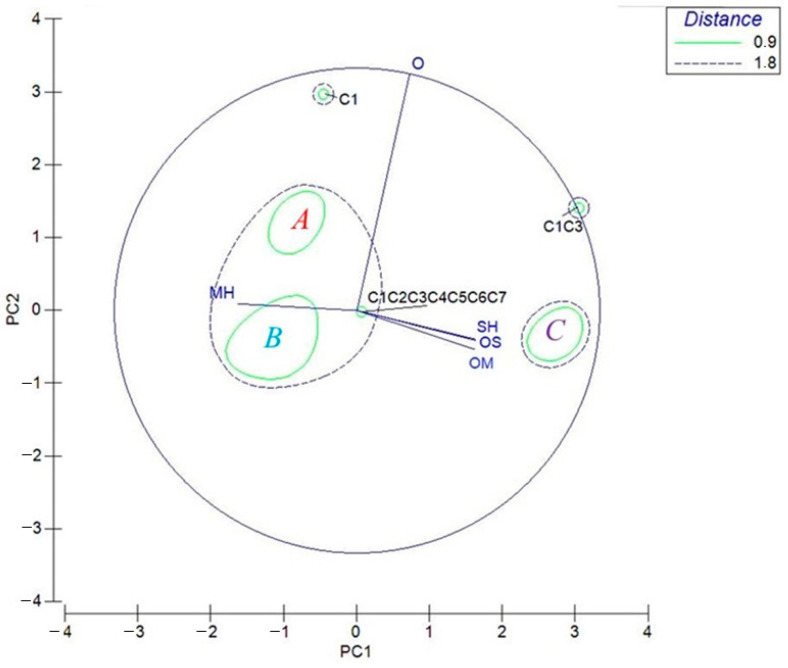
Principal component analysis (PCA) of EOs from *C. aurantium* and their mixes based on the principal classes of compounds: monoterpene hydrocarbons (MH), oxygenated monoterpenes (MO), sesquiterpenes hydrocarbons (SH), oxygenated sesquiterpenes (OS), and others (O). The vectors shown are the eigenvectors of the covariance matrix. Group ‘A’ included C1C2, C1C4, C1C5, C1C6, and C1C7 samples; group ‘B’ contained the samples C2, C4, C5, C6, C7, C2C4, C2C5, C2C6, C2C7, C4C5, C4C6, C4C7, C5C6, C5C7, and C6C7; group ‘C’ included C2C3, C3C4, C3C5, C3C6, and C3C7 samples.

For the EOs of the different *C. aurantium* cultivars and their mixes, as shown in the loading graph (Figure 2), all variables influenced PC1 and PC2. In fact, PC1 (81.8% of the total significant contribution) was represented mainly by oxygenated monoterpenes and sesquiterpenes (OM and OS, respectively), by sesquiterpene hydrocarbons (SH), and in a minor contribution by O in the positive score, and by MH eigenvalue in negative scores; meanwhile, PC2 (17.1%) was represented mainly by a positive score of O compounds.

**Figure 3:**
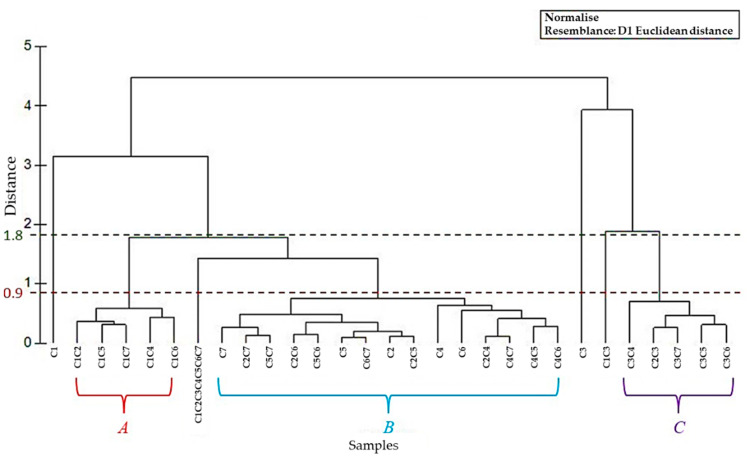
Dendrogram obtained by hierarchical cluster analysis (HCA) based on the Euclidian distances between groups of A, B, and C, for *C. aurantium* EOs and their mixes. The samples’ codes are reported in Section 3.1.

HCA, based on the Euclidean distance between groups, indicated three different clusters (‘A’, ‘B’, and ‘C’, Figure 3), considering the chemotaxonomic similarity < 0.9.

In the first group, ‘A’, from the HCA analysis, samples C1C2, C1C4, C1C5, C1C6, and C1C7 were included, whose monoterpene hydrocarbons content varied between 85.29 and 91.70%. This cluster was distinguished from cluster ‘B’ by the higher quantity of other compounds (O), such as *n*-octanal (1.68–2.53%) and *n*-decanal (0.77–1.08%).

A definite macro-group ‘B’, containing the samples C2, C4, C5, C6, C7, C2C4, C2C5, C2C6, C2C7, C4C5, C4C6, C4C7, C5C6, C5C7, and C6C7, was characterized by single EO and some mixes with a large percentage of monoterpene hydrocarbons (84.36–97.41%), by the absence of both oxygenated and hydrocarbon sesquiterpenes, and by the minimal (maximum 1.35% only for C4 EO) presence and/or absence of compounds belonging to the O class. Finally, the C2C3, C3C4, C3C5, C3C6, and C3C7 samples can be grouped in cluster ‘C’, due to the influence of the C3 EO. This cluster was characterized by a good quantity of MH (59.91–67.95%), but, compared to the other two groups ‘A’ and ‘B’, it had a moderate amount of OM (22.42–27.13%), and a small presence of SH and OS (2.13–2.33% and 2.25–2.33%, respectively). Sample C1C3 cannot be included in this ‘C’ cluster due to the content—not excessive, but significant—of other compounds (O) (4.64%, compared to the range 1.53–2.36% in the other samples). Furthermore, the choice of a cut-off of 1.8 would have significantly decreased the similarity between the samples analyzed, leading to an incorrect grouping. The others two samples, C1 and C3—which were first for the significant quantity of other compounds (5.84%), and second for the greatest presence of OM (44.79%) and MH (36.21%), respectively—were considered as two separate classes, without any similarity with the other clusters. On the other hand, the mix C1C2C3C4C5C6C7, can be considered as an intermediate sample between clusters ‘A’ and ‘B’, but with small characteristics (the content of SH, OS, and OM) referred to cluster ‘C’.

### 2.3. Antioxidant Activity

The antioxidant activity of seven *C. aurantium* flavedo EOs and their mixes were investigated using different tests. DPPH and ABTS are based on an electronic transfer reaction, whereas β-carotene bleaching inhibition test and FRAP are based on a transfer reaction of a hydrogen atom. In all assays except the FRAP test, EOs and their mixes exhibited antioxidant activity in a concentration-dependent manner. Among tested EOs, *C. aurantium* ‘Consolei’ (C2) showed the highest DPPH radical scavenging potential, with IC_50_ value of 33.01 μg/mL, followed by flavedo EO from cultivar ‘Fasciata’ (C4, IC_50_ value of 33.98 μg/mL), whereas sample *C. aurantium* ‘Canaliculata’ (C1) exhibited the highest ABTS radical scavenging ability, with IC_50_ value of 25.31 μg/mL. A promising ABTS radical scavenging potential was observed also with EO C6 and C4 (IC_50_ values of 27.38 and 27.45 μg/mL, respectively) (Table 2). The same samples are also the most active in protection from lipid peroxidation, with IC_50_ values of 15.46 and 18.56 μg/mL for C4 and C6, respectively. EOs obtained from ‘Fasciata’ and ‘Listata’ cultivar have FRAP values comparable to the positive control BHT (55.92 and 50.23 μM Fe^2+^/g for C3 and C5, respectively, vs 63.27 μM Fe^2+^/g).

As for the oil combinations obtained by mixing the EOs in the ratio 1:1 (*v*/*v*), the C1C6 sample showed the highest potential to inactivate the DPPH radical, with IC_50_ value of 31.05 μg/mL, followed by the C1C7 sample (IC_50_ value of 31.44 μg/mL). A similar DPPH radical scavenging activity was observed also with C1C4 and C3C6. Combination of EO from *C. aurantium* cultivars ‘Foetifera’+‘Bizzaria’ (C5C7) and from ‘Crispifolia’ + ‘Bizzaria’ (C3C7) exhibited a promising ABTS radical scavenging potential, with IC_50_ values of 31.12 and 31.40 μg/mL, respectively. In the β-carotene bleaching test, combination C1C7 was the most active, with an IC_50_ value of 13.42 μg/mL, followed by C2C6 and C4C7. Moreover, we have no evidence of significant differences between EOs tested alone and their combinations in the reduction of iron, with values ranging from 19.98 to 38.06 μM Fe^2+^/g for C1C2 and C1 + C2 + C3 + C4 + C5 + C6 + C7, respectively.

Several studies have demonstrated that *Citrus* flavedo is a promising source of antioxidant compounds. This means that, even if present in low concentration, EO constituents are characterized by a certain antioxidant activity. Moreover, the different activity of the oil should be explained not only by the different geographical origin but also from the extraction procedure applied to obtain the oil; both factors, in fact, affect the composition of the oils. Sarrou et al. [15] investigated the different DPPH radical scavenging potentials of *C. aurantium* flavedo, flowers, and young and old leaf EO from samples growing in Greece and found that flavedo oil is characterized by the lowest activity, with a percentage of inhibition of 19.29%.

Previously, our research group investigated the antioxidant activity of *C. aurantifolia*, *C. aurantium*, and *C. bergamia* flavedo EOs from plant collected in Calabria (South of Italy) [30]. In this case, *C. aurantium* sample showed a lower DPPH radical scavenging potential (IC_50_ value of 188.9 μg/mL), whereas the protection from lipid peroxidation is quite similar (IC_50_ value of 55.7 μg/mL). The effect of the extraction procedure on the antioxidant activity was studied by Lu et al. [32], who tested the DPPH radical scavenging activity of *Citrus* flavedo oils extracted by cold pressing and hydro-distillation. EOs obtained by hydro-distillation process are characterized by a greater activity, with EC_50_ values of 41.68 and 51.44 μL/mL vs. 133.57 and 77.22 μL/mL for *C. sinensis* Valencia and *C. reticulata* Blanco, respectively.

A lower DPPH radicals scavenging potential was observed by Kamal et al. [33] with *C. paradisi*, *C. sinensis*, and *C. reticulata* flavedo EOs, with percentage of inhibition ranging from 14.05 to 24.08% when tested at a concentration of 100 μg/mL. The same oils are able to inhibit the oxidation of linoleic acid, with percentage of inhibition ranging from 54.98 to 67.80% for *C. reticulata* and *C. sinensis*, respectively. d-limonene was the main abundant compound in all investigated EOs. Previously, Shah and Mehta [34] demonstrated that d-limonene exerted an appreciable radical scavenging activity in both ABTS and DPPH tests, with IC_50_ values of 603.23 and 384.73 µM, respectively. A comparable Trolox-reducing ferric ability was also observed.

The global antioxidant score (GAS) allows one to discriminate the matrix with the best antioxidant power by combining the differentiated results of a large antioxidant battery (Figure S1). Based on the data, *C. aurantium* ‘Fasciata’ EO (C4) exhibited the highest antioxidant potential. Among combinations of EOs samples, C1C7 and C5C7 were the most active.

## 3. Materials and Methods

### 3.1. Plant Material

The seven *C. aurantium* cultivars analyzed—preserved in the Botanical Garden of Palermo (38°06′48.39″ N; 13°22′21.68″ E), Sicily, and collected in January 2020—are those that were reported by Riccobono [35] as *C*. *aurantium* ‘Canaliculata’ (C1), *C. aurantium* ‘Consolei’ (C2), *C*. *aurantium* ‘Crispifolia’ Risso & Poit. (C3), *C*. *aurantium* ‘Fasciata’ Risso & Poit. (C4), *C*. *aurantium* ‘Foetifera’ (C5), *C*. *aurantium* ‘Listata’ (C6), and *C. aurantium* ‘Bizzaria’ (C7). The samples, identified by Prof. Rosario Schicchi and Prof. Anna Geraci, were kept in the Herbarium Mediterraneum of the Botanical Garden of the University of Palermo (PAL). The number of the voucher is reported for each cultivar. *C. aurantium* ‘Canaliculata’ (Voucher No. 109736) has lemon-like leaves and fruits with aureus-rubescent tuberculate epicarp; thick, spongy, flavorful mesocarp; and juicy, acidic flesh [36]. *C. aurantium* ‘Consolei’ (Voucher No. 109737) is characterized by fruits of intense orange color, ribbed and with wide and deep longitudinal grooves; *C. aurantium* ‘Crispifolia’ (Voucher No. 109738) is known by the local name “*Aranciu amaru a fogghi rizzi*” (sour orange with curled leaves) because it has oval-oblong, curled, dense leaves and small fruits; C. *aurantium* ‘Fasciata’ (Voucher No. 109739) has fruits with raised green longitudinal stripes and deeper yellow stripes, which turn orange and chrome-yellow at maturity, respectively; *C. aurantium* ‘Foetifera’ (Voucher No. 109740) has reflexed leaves and large, flattened fruits, characterized in the apical part by the presence of a small, more or less well-formed recessed fruit called a “fetus”; and *C. aurantium* ‘Listata’ (Voucher No. 109741) has variegated leaves and fruits with a listate epicarp, but this characteristic is lost almost completely during full maturity. *C. aurantium* ‘Bizzaria’ (Voucher No. 109735) is a *Citrus* fruit that shares its name with a chimera that has characteristics intermediate between the bitter orange (*C. aurantium*) and the citron (*C. medica* L.) [37]. The cultivar under examination has characteristics only of bitter orange. It presents small, spherical fruits, having in the apical part an evident circular sculpture.

### 3.2. Essential Oil Extraction and Their Mix Preparation

Extraction of EOs was carried out according to Catinella et al. [38]. A variable quantity of flavedo, the colored outer layer of the rind of a *Citrus* fruit, obtained by peeling the fruits with an electric peeler (093209-006-BLCK, 770 Boulevard Guimond, Longueuil Quebec, Canada) apparatus, of C1 (117 g), C2 (130 g), C3 (111 g), C4 (71 g), C5 (159 g), C6 (111 g), and C7 (122 g) was subjected to hydro-distillation for 3 h using Clevenger’s apparatus [39]. The oil yields were 1.82, 1.37, 2.28, 1.93, 2.35, 2.19, and 1.36% (*v*/*w*), respectively. Combinations of EOs were obtained by mixing oils in a 1:1 *v*/*v* ratio. EOs and their mixes were stored in the freezer at −20 °C, until the time of analyses.

### 3.3. GC-MS Analysis of EOs

Analyses of EOs and their mixes were performed according to the procedure reported by Basile et al. [40]. EO analysis was performed using an Agilent 7000 C GC (Agilent Technologies, Inc., Santa Clara, CA, USA) system, fitted with a fused silica Agilent DB-5 MS capillary column (30 m × 0.25 mm i.d.; 0.25 μm film thickness) and coupled to an Agilent triple quadrupole mass selective detector MSD 5973 (Agilent Technologies, Inc., Santa Clara, CA, USA). The settings were as follows: ionization voltage, 70 eV; electron multiplier energy, 2000 V; transfer line temperature, 295 °C; solvent delay, 4 min. The oven program was as follows: temperature increase at 40 °C for 5 min at a rate of 2 °C/min up to 260 °C, and then isothermal amplification for 20 min. Helium was used as the carrier gas (1 mL/min). The injector and detector temperatures were set at 250 °C and 290 °C, respectively. An amount of 1 μL of each EO solution (3% EO/hexane *v*/*v*) was injected with a split mode. Chromatograms of all EOs are reported in Supplementary Material (Figures S2–S8). Linear retention indices (LRI) were determined by using retention times of *n*-alkanes (C_8_-C_40_), and the peaks were identified by comparison with mass spectra and by comparison to their relative retention indices with WILEY275 (Wiley), NIST 17 (NIST, The National Institute of Standards and Technology, Gaithersburg, MD, USA) [41], and FFNSC2 (Shimadzu, Kyoto, Japan) libraries.

### 3.4. Antioxidant Activity

#### 3.4.1. Evaluation of Radical Scavenging Activity by ABTS and DPPH Assay

*Citrus aurantium* EOs and their mixes were screened for their radical scavenging activity by using 2,2′-azino-bis-3-ethylbenzthiazoline-6-sulphonic acid (ABTS) and 1,1-diphenyl-2-picrylhydrazyl (DPPH) tests [42]. Briefly, a solution of ABTS radical was obtained by mixing 7 mM ABTS solution with 2.45 mM K_2_S_2_O_8_. After one night, the solution was diluted with EtOH until an absorbance of 0.70 at 734 nm using a UV-Vis Jenway 6003 spectrophotometer (Jenway, Mortdale, Australia) was read. Dilutions of samples were added to diluted ABTS radical solution to test concentrations from 400 to 1 μg/mL. After 6 min of incubation, the absorbance was read at 734 nm. In DPPH radical scavenging assay, a mixture of 0.25 mM DPPH radical (DPPH·) in ethanol and EOs were prepared to test concentrations ranging from 1000 to 1 μg/mL. The mixture constituted by EO/mix and DPPH radical solution was vigorously shaken and left to react at 25 °C for 30 min. The discoloration of the DPPH was determined at 517 nm. Ascorbic acid was used as a positive control in both tests.

#### 3.4.2. Ferric Reducing Ability Power (FRAP Assay)

FRAP test is based on the reaction that involves 2,4,6-tripyridyl-s-triazine-Fe^3+^ complex (TPTZ/Fe^3+^). Briefly, a mixture namely FRAP reagent, consisting of 40 mM HCl, 20 mM FeCl_3_, and 0.3 M acetate buffer at pH 3.6, was prepared [42]. EO at concentration of 2.5 mg/mL in MeOH (100 μL) was mixed with FRAP reagent and water. To monitor the reduction of TPTZ/Fe^3+^ to TPTZ/Fe^2+^, the absorption was measured at 595 nm after 30 min of incubation at room temperature. A calibration curve was obtained by using ethanol solutions of known Fe^2+^ concentration, in the range of 50–500 μM. The FRAP value was expressed as μM Fe^2+^/g. Butylated hydroxytoluene (BHT) was used as a positive control.

#### 3.4.3. Carotene Bleaching Test

The capacity of the EOs and their combinations to inhibit lipid peroxidation in both initiation and propagation phase was studied by using the β-carotene bleaching test [42].

In this assay, a mixture of β-carotene, linoleic acid, and Tween 20 was prepared. After solvent evaporation and dilution with water, the emulsion was added to a 96-well microplate containing EOs (12 μL) at final concentrations ranging from 100 to 2.5 μg/mL. The microplate was shaken and left to incubate at 45 °C for 60 min. After that, the absorbance was read at 470 nm. Propyl gallate was used as a positive control.

### 3.5. Statistical Analysis

All analyses were performed in triplicate. Data are expressed as means ± standard deviation (S.D.). The concentration–response curve was obtained by plotting the percentage inhibition vs. concentration. The concentration that yielded 50% inhibition (IC_50_) was calculated by nonlinear regression with the use of Prism GraphPad Prism version 4.0 for Windows (GraphPad Software, San Diego, CA, USA). Tukey’s test was applied to determine any significant difference in chemical parameters among investigated samples (** *p* < 0.05).

To get a ranking of EOs and their mixes’ antioxidant capacity, global antioxidant score (GAS) was calculated. This statistical application was generated by integrating the antioxidant capacity values generated from different in vitro methods. The mean of 3 T-scores was taken for the value of GAS, ranging from 0 to 3. T-score is calculated by the following equation:

T-s c or e = (X − min)/(max − min)

where min and max, respectively, represent the smallest and largest values of variable X among the antioxidant values of the same EO [43].

Principal component analysis was performed according to the procedure reported by Bancheva et al. [44]. The different chemical classes used to describe the composition of individual essential oils and their mixes were considered as original variables and subjected, after normalization, to cluster analysis (CA) and to principal component analysis (PCA). The statistical analyses were performed using PRIMER 6 (Massey University Eastbourne, Albany, New Zealand) with two principal components (PC) variables, and the number of clusters was determined by using the rescaled distances in the dendrogram, using a cut-off point (Euclidean distance = 0.9) that allows the attainment of consistent clusters. The principal components analysis (PCA) and the hierarchical cluster analysis (HCA) were used to comprehend the similarities among the essential oils in relation to the contents of their chemical constituents. We tested two different cut-off similarity levels (cut-off level 0.9 and cut-off level 1.8), chosen based on the mean distance between clusters and based on the similarities–differences between the samples belonging to the same cluster. Since the HCA analysis is a function of variables and observations, the highest correspondence between PCA and HCA resulted when we applied a cut-off of 0.9. The statistical analysis of the absence/presence was carried out using the cluster method of the PRIMER 6 software (Massey University Eastbourne, Albany, New Zealand) [45].

## 4. Conclusions

*Citrus* flavedo EOs have been extensively studied and widely used in pharmaceutical, food, and cosmetic industries, and are generally recognized as safe (GRAS). Herein, EOs obtained by hydrodistillation of the flavedo of *C. aurantium* cultivars ‘Canaliculata’, ‘Consolei’, ‘Crispifolia’, ‘Fasciata’, ‘Foetifera’, ‘Listata’, and ‘Bizzaria’ collected in Sicily were investigated alone and as combinations. d-limonene was the main abundant monoterpene hydrocarbon. Chemical and statistical analyses (PCA and HCA) can provide chemodiversity information on the investigated samples. A division of the cultivars into two classes emerged: the first one, composed of *C. aurantium* C1, C2, C4, C5, C6, and C7 samples, was characterized by a high amount of d-limonene, and a separate cultivar, C3, contained a lesser quantity of d-limonene but higher percentage of oxygenated monoterpenes. On the other hand, more detailed PCA and HCA analyses showed the clustering of EOs into three groups: group ‘A’, with a high monoterpene hydrocarbons content and a good quantity of (O); a definite group ‘B’, characterized by a large percentage of MH, by the absence of SH and OS, and by the minimal presence of O class; and finally, cluster ’C’, showing a moderate amount of OM and a small presence of SH and OS. All EOs are able to exert antioxidant activity via different mechanisms of action, as revealed by different applied tests. Among them, the *C. aurantium* ‘Fasciata’ EO showed the highest antioxidant potential, whereas among combinations, a promising antioxidant activity was observed with the sample obtained by mixing, in equal volumes, cultivar ‘Canaliculata’ + ‘Bizzaria’. According to Ambrosio et al. [46], the founded bioactivity is not linked to the main abundant compound d-limonene. Therefore, our results support the importance of the involvement of minor compounds in the antioxidant activity of the samples. Moreover, the comparison between EOs combinations’ inhibitory concentration (IC_50_) and FRAP values’ theoretical calculation (data not shown) and real data did not clearly highlight phenomena of synergism or antagonism of action valid in all biological tests.

## Figures and Tables

**Table 1:** Composition (%) of the essential oils of the seven *C. aurantium* cultivars collected in the Botanical Garden of Palermo, Sicily.

						Content (%) ^C^						
No.	Compounds	LRI_exp_^A^	LRI_lit_^B^	C1	C2	C3	C4	C5	C6	C7	Ident. ^D^	Sign. ^E^
1	α-Pinene	936	934	2.98 ^a^	2.57 ^c^	-	2.69 ^b^	1.67 ^d^	3.01 ^a^	1.49 ^e^	1, 2, 3	**
2	β-Pinene	977	981	3.33 ^a^	-	0.91 ^d^	2.71 ^b^	-	2.73 ^b^	1.58 ^c^	1, 2, 3	**
3	β-Myrcene	990	994	2.76 ^d^	7.52 ^a^	1.95 ^f^	2.04 ^e^	5.61 ^b^	1.00	4.13 ^c^	1, 2, 3	**
4	*n*-Octanal	1001	1005	3.77 ^a^	-	-	1.35 ^b^	-	-	-	1, 2	**
5	d-limonene	1031	1028	77.53 ^d^	80.50 ^c^	33.35 ^f^	76.00 ^e^	82.78 ^b^	76.06 ^e^	89.17 ^a^	1, 2, 3	**
6	β-*cis*-Ocimene	1038	1036	-	1.45 ^a^	-	0.92 ^d^	-	1.18 ^c^	1.04 ^b^	1, 2	**
7	*n*-Octanol	1070	1078	-	-	1.59 ^a^	-	-	-	-	1, 2, 3	**
8	β-Linalool	1098	1101	2.58 ^c^	1.04 ^f^	7.69 ^a^	2.67 ^b^	1.89 ^e^	2.31 ^d^	1.03 ^f^	1, 2	**
9	α-Terpineol	1189	1194	-	-	7.06 ^a^	1.08 ^c^	0.91 ^d^	1.17 ^b^	-	1, 2	**
10	*n*-Decanal	1203	1208	2.07 ^a^	-	-	-	-	-	-	1, 2	**
11	*cis*-Geraniol	1229	1235	-	-	1.63 ^a^	-	-	-	-	1, 2	**
12	β-Citral	1238	1242	-	-	1.88 ^a^	-	-	-	-	1, 2	**
13	Bergamol	1256	1258	-	1.46 ^d^	6.77 ^a^	3.55 ^c^	1.43 ^d^	4.33 ^b^	-	1, 2	**
14	*trans*-Geraniol	1259	1267	-	-	3.36 ^a^	-	-	-	-	1, 2	**
15	Neryl Acetate	1365	1366	-	-	6.28 ^a^	-	-	-	-	1, 2	**
16	Geranyl Acetate	1386	1392	-	-	10.12 ^a^	1.41 ^c^	-	1.51 ^d^	-	1, 2	**
17	*n*-Decyl Acetate	1402	1406	-	-	1.44 ^a^	-	-	-	-	1, 2	**
18	Caryophyllene	1419	1423	-	-	1.00 ^a^	-	-	-	-	1, 2, 3	**
19	Germacrene D	1480	1485	-	-	3.48 ^a^	-	-	-	-	1, 2	**
20	*trans*-Nerolidol	1550	1554	-	-	4.57 ^a^	-	-	-	-	1, 2	**
Monoterpene Hydrocarbons				86.60	92.04	36.21	84.36	90.06	84.52	97.41		
Oxygenated Monoterpenes				2.58	2.50	44.79	8.71	4.23	9.32	1.03		
Sesquiterpene Hydrocarbons				-	-	4.48	-	-	-	-		
Oxygenated Sesquiterpenes				-	-	4.57	-	-	-	-		
Others				5.84	-	3.03	1.35	-	-	-		
	Total			95.02	94.54	93.08	94.42	94.29	93.84	98.44		

^A^ Linear retention index, obtained through the modulated chromatogram, reported for DB-5MS apolar column; ^B^ linear retention index reported for DB-5MS column or equivalents reported in the literature; ^C^ content is the peak volume percentage of compounds in the essential oil sample; ^D^ 1 = retention index identical to bibliography; 2 = identification based on comparison of MS; 3 = retention time identical to authentic compounds. Compounds are classified in order of linear retention time of non-polar column. ^E^ Sign: significance at *p* < 0.05. Results followed by different letters in the same line are significantly different (*p* < 0.05) according to Tukey’s multiple range test.

**Table 2:** Antioxidant activities of seven *C. aurantium* cultivars EO and their combinations.

	DPPH (IC_50_ μg/mL)	ABTS (IC_50_ μg/mL)	β-Carotene Bleaching Test (IC_50_ μg/mL)	FRAP μM Fe^2+^/g
*C. aurantium* EO				
C1	38.39 ± 2.12 ^g^	25.31 ± 2.66 ^a^	38.61 ± 3.54 ^n^	20.62 ± 2.36 ^n^
C2	33.01 ± 1.71 ^c^	30.14 ± 2.27 ^d^	22.68 ± 2.16 ^g^	28.23 ± 4.12 ^h^
C3	40.18 ± 2.82 ^i^	36.22 ± 2.42 ^j^	55.72 ± 3.87 ^v^	55.92 ± 2.92 ^a^
C4	33.98 ± 2.00 ^c^	27.45 ± 1.85 ^b^	15.46 ± 1.31 ^b^	44.44 ± 3.66 ^d^
C5	39.01 ± 2.74 ^h^	29.56 ± 2.93 ^c^	48.73 ± 2.64 ^q^	50.23 ± 3.41 ^b^
C6	37.93 ± 2.54 ^f^	27.38 ± 1.77 ^b^	18.56 ± 1.56 ^d^	20.65 ± 1.78 ^n^
C7	40.74 ± 3.18 ^i^	32.32 ± 2.72 ^f^	49.68 ± 2.93 ^r^	45.44 ± 2.45 ^c^
EOs Combination (1:1 *v*/*v*)				
C1C2	36.12 ± 1.81 ^e^	38.39 ± 2.75 ^l^	57.28 ± 3.41 ^z^	19.98 ± 1.98 ^o^
C1C3	42.09 ± 2.48 ^k^	33.01 ± 2.84 ^g^	25.77 ± 2.58 ^j^	21.72 ± 1.92 ^m^
C1C4	31.75 ± 2.33 ^a^	40.18 ± 3.01 ^n^	50.63 ± 3.67 ^s^	23.08 ± 2.01 ^k^
C1C5	32.64 ± 2.54 ^b^	33.98 ± 2.52 ^g^	31.96 ± 2.73 ^k^	27.18 ± 2.23 ^i^
C1C6	31.05 ± 2.25 ^a^	39.01 ± 2.96 ^m^	53.82 ± 3.86	20.73 ± 2.14 ^m^
C1C7	31.44 ± 2.63 ^a^	37.93 ± 2.84 ^kl^	13.42 ± 2.24 ^a^	22.60 ± 2.35 ^l^
C2C3	37.16 ± 2.85 ^f^	39.42 ± 2.73	41.14 ± 3.72 ^p^	22.04 ± 2.32 ^l^
C2C4	34.60 ± 2.16 ^d^	35.16 ± 2.4 ^i^	24.12 ± 1.94 ^i^	23.49 ± 2.41 ^k^
C2C5	38.49 ± 2.92 ^g^	36.85 ± 2.64 ^j^	37.66 ± 2.57 ^m^	24.26 ± 2.66 ^j^
C2C6	38.68 ± 2.73 ^g^	33.98 ± 2.25 ^g^	17.53 ± 2.01 ^c^	22.55 ± 2.47 ^l^
C2C7	37.60 ± 2.77 ^f^	38.13 ± 2.74 ^l^	25.95 ± 2.43 ^j^	23.14 ± 2.52 ^k^
C3C4	41.96 ± 3.24 ^j^	46.23 ± 3.88 ^o^	22.68 ± 2.52 ^g^	22.91 ± 2.36 ^l^
C3C5	33.91 ± 2.45 ^c^	37.16 ± 3.57 ^k^	55.06 ± 3.04 ^v^	22.71 ± 2.2 ^l^
C3C6	31.86 ± 2.24 ^a^	33.08 ± 2.35 ^g^	36.08 ± 2.86 ^l^	23.11 ± 2.31 ^k^
C3C7	36.37 ± 2.57 ^e^	31.40 ± 2.49 ^e^	37.41 ± 2.97 ^m^	23.19 ± 2.47 ^k^
C4C5	34.38 ± 2.35 ^d^	36.22 ± 2.71 ^j^	23.54 ± 2.03 ^h^	29.61 ± 3.05 ^g^
C4C6	37.16 ± 2.76 ^f^	34.68 ± 2.52 ^h^	57.72 ± 3.81 ^z^	30.95 ± 3.16 ^f^
C4C7	34.60 ± 2.40 ^d^	38.51 ± 2.73 ^l^	19.54 ± 1.61 ^e^	27.23 ± 2.42 ^i^
C5C6	38.49 ± 3.48 ^g^	38.34 ± 2.65 ^l^	54.86 ± 3.33 ^u^	28.06 ± 2.77 ^h^
C5C7	38.68 ± 3.39 ^g^	31.12 ± 2.44 ^e^	21.64 ± 1.57 ^f^	30.97 ± 3.26 ^f^
C6C7	37.60 ± 2.98 ^f^	34.30 ± 2.25 ^h^	51.23 ± 3.58 ^t^	30.14 ± 3.01 ^f^
C1 + C2 + C3 + C4 + C5 + C6 + C7	44.96 ± 3.70 ^l^	39.23 ± 2.73 ^m^	40.21 ± 3.29 ^o^	38.06 ± 3.54 ^e^
Sign	**	**	**	**

Data are expressed as means ± standard deviation (SD). The following positive controls were used: ascorbic acid in 2,2-Diphenyl-1-picrylhydrazyl (DPPH) (IC_50_ value of 5.03 ± 0.79 μg/mL) and 2,2′-Azino-Bis-3-Ethylbenzothiazoline-6-Sulfonic acid (ABTS) (IC_50_ value of 1.72 ± 0.09 μg/mL) test; propyl gallate in β-carotene bleaching test (IC_50_ value of 0.09 ± 0.004 μg/mL); butylated hydroxytoluene (BHT) in ferric reducing ability power (FRAP) (FRAP value 63.27 ± 4.48 μM Fe(II)/g). Sign: significance at ** *p* < 0.05. Results followed by different letters in the same line are significantly different according to Tukey’s multiple range test.

## Data Availability

All data and materials are available on request to the corresponding author.

## Supplementary Materials

The following supporting information can be downloaded. Figure S1: Comparative global antioxidant score (GAS) of *C. aurantium* cultivars EO and their combinations. Table S1: Composition (%) of mixes of the essential oils. Figure S2: Chromatogram obtained by injection of *C. aurantium* ‘Canaliculata’ (C1) EO. Figure S3. Chromatogram obtained by injection of *C. aurantium* ‘Consolei’ (C2) EO. Figure S4. Chromatogram obtained by injection of *C. aurantium* ‘Crispifolia’ (C3) EO. Figure S5. Chromatogram obtained by injection of *C. aurantium* ‘Fasciata’ (C4) EO. Figure S6. Chromatogram obtained by injection of *C. aurantium* ‘Foetifera’ (C5) EO. Figure S7. Chromatogram obtained by injection of *C. aurantium* ‘Listata’ (C6) EO. Figure S8. Chromatogram obtained by injection of *C. aurantium* ‘Bizzaria’ (C7) EO.

## Sample Availability

Samples of *C. aurantium* oils are available from the authors.
