# Supplementary material for: Chemical Compositions and Antioxidant Activities of Essential Oils, and Their Combinations, Obtained from Flavedo By-Product of Seven Cultivars of Sicilian Citrus aurantium L
Source: Molecules. 2022 Feb 27;27(5):1580. doi: 10.3390/molecules27051580 (PMC8911714; doi:10.3390/molecules27051580)
Supplement: Supplementary file 1 [file molecules-27-01580-s001.zip › molecules-1612833-supplementary.pdf]

Supplementary material

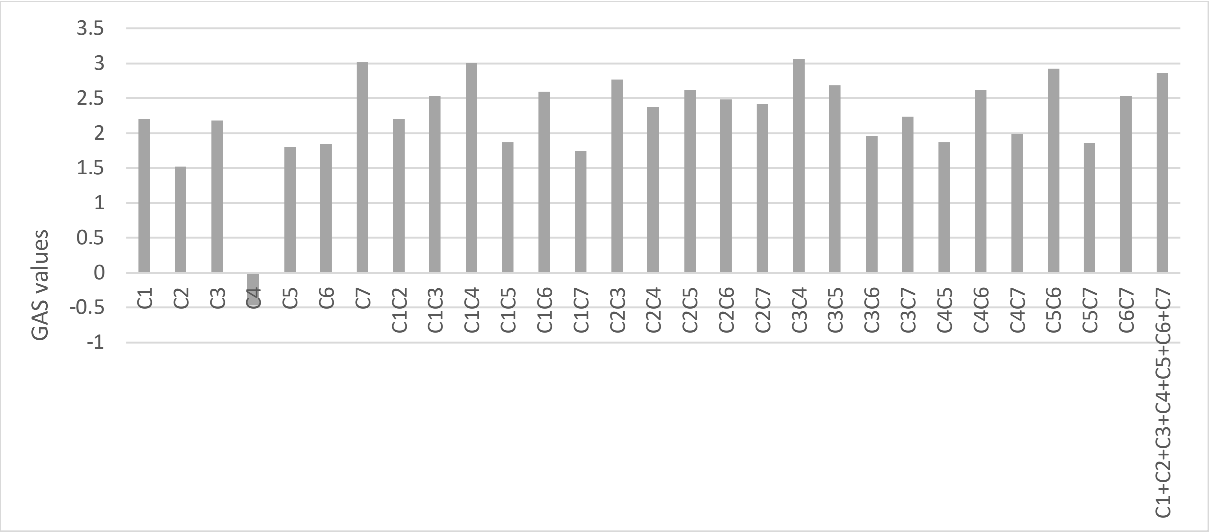

Figure S1. Comparative Global Antioxidant Score (GAS) of *C. aurantium* cultivars EO and their combinations

Table S1. Composition (%) of mixes of the essential oils.

Compounds	C1C2	C1C3	C1C4	C1C5	C1C6	C1C7	C2C3	C2C4	C2C5	C2C6	C2C7
α-Pinene	2.78	1.42	2.84	2.33	3.00	2.19	1.23	2.58	2.08	2.71	2.00
β-Pinene	1.72	2.13	3.00	1.70	2.98	2.42	0.53	1.41	-	1.44	0.83
β-Myrcene	5.14	2.34	2.39	4.18	1.89	3.29	4.76	4.81	6.66	4.32	5.88
n-Octanal	1.68	1.93	2.53	1.95	1.88	1.85	-	0.66	-	-	-
D-Limonene	79.02	55.40	76.75	80.15	76.82	83.21	60.01	78.50	81.74	78.44	84.83
β-*cis*-Ocimene	0.76	-	0.54	-	0.60	0.59	0.68	1.32	0.69	1.33	1.29
n-Octanol	-	0.84	-	-	-	-	0.83	-	-	-	-
β-Linalool	1.84	5.11	2.63	2.21	2.43	1.75	4.38	1.88	1.48	1.58	1.00
α-Terpineol	-	3.72	0.48	0.45	0.56	-	3.56	0.57	0.50	0.55	-
n-Decanal	0.77	1.09	1.07	1.08	1.05	1.03	-	-	-	-	-
cis-Geraniol	-	0.82	-	-	-	-	0.83	-	-	-	-
β-Citral	-	0.97	-	-	-	-	1.01	-	-	-	-
Bergamol	0.68	3.28	1.80	0.80	2.12	-	4.05	2.38	1.43	2.92	0.78
trans-Geraniol	-	1.74	-	-	-	-	1.72	-	-	-	-
Neryl acetate	-	3.31	-	-	-	-	3.29	-	-	-	-
Geranyl acetate	-	5.11	0.69	-	0.68	-	5.18	0.77	-	0.70	-
n-Decyl acetate	-	0.78	-	-	-	-	0.74	-	-	-	-
Caryophyllene	-	0.49	-	-	-	-	0.54	-	-	-	-
Germacrene D	-	1.65	-	-	-	-	1.63	-	-	-	-
trans-Nerolidol	-	2.25	-	-	-	-	2.25	-	-	-	-
Monoterpene Hydrocarbons	89.42	61.29	85.52	88.36	85.29	91.70	67.21	88.62	91.17	88.24	94.83
Oxygenated Monoterpenes	2.52	24.06	5.60	3.46	5.79	1.75	24.02	5.60	3.41	5.75	1.78
Sesquiterpene Hydrocarbons	-	2.14	-	-	-	-	2.17	-	-	-	-
Oxygenated Sesquiterpenes	-	2.25	-	-	-	-	2.25	-	-	-	-
Others	2.45	4.64	3.60	3.03	2.93	2.88	1.57	0.66	-	-	-

Total	94.39	94.38	94.72	94.85	94.01	96.33	97.22	94.88	94.58	93.99	96.61
continued…											

Compounds	C3C4	C3C5	C3C6	C3C7	C4C5	C4C6	C4C7	C5C6	C5C7	C6C7	mix*
α-Pinene	1.44	0.91	2.89	0.78	2.20	2.98	2.16	2.30	1.64	2.18	2.11
β-Pinene	1.67	0.55	1.85	1.27	1.33	2.74	2.17	1.44	0.80	2.19	1.54
β-Myrcene	2.00	3.45	1.62	3.13	3.90	1.56	3.03	3.35	4.75	2.30	3.53
n-Octanal	0.84	-	-	-	0.53	0.67	0.68	-	-	-	0.70
D-Limonene	54.32	58.04	54.99	62.17	80.01	76.13	81.47	79.23	85.78	82.56	72.99
β-*cis*-Ocimene	0.48	-	0.59	0.60	0.43	1.03	0.95	0.60	0.56	1.11	0.62
n-Octanol	0.83	0.83	0.80	0.77	-	-	-	-	-	-	0.25
β-Linalool	5.23	4.35	4.99	4.19	2.19	2.50	1.87	2.08	1.32	1.58	2.65
α-Terpineol	4.18	3.99	4.08	3.49	1.03	1.18	0.56	1.03	0.56	0.66	1.60
n-Decanal	-	-	-	-	-	-	-	-	-	-	0.31
cis-Geraniol	0.82	0.84	0.88	0.89	-	-	-	-	-	-	0.23
β-Citral	1.05	1.00	0.99	1.01	-	-	-	-	-	-	0.29
Bergamol	5.12	4.08	5.53	3.22	4.38	3.84	1.73	2.94	0.68	2.12	2.51
trans-Geraniol	1.69	1.67	1.57	1.62	-	-	-	-	-	-	0.45
Neryl acetate	3.27	3.28	3.03	2.89	-	-	-	-	-	-	0.90
Geranyl acetate	5.79	5.14	5.67	5.11	0.66	1.42	0.69	0.72	-	0.69	1.74
n-Decyl acetate	0.69	0.70	0.82	0.88	-	-	-	-	-	-	0.19
Caryophyllene	0.56	0.55	0.49	0.51	-	-	-	-	-	-	0.16
Germacrene D	1.62	1.58	1.84	1.87	-	-	-	-	-	-	0.55
trans-Nerolidol	2.28	2.27	2.30	2.33	-	-	-	-	-	-	0.67
Monoterpene Hydrocarbons	59.91	62.95	61.94	67.95	87.87	84.44	89.78	86.92	93.53	90.34	80.79
Oxygenated Monoterpenes	27.15	24.35	26.74	22.42	8.26	8.94	4.85	6.77	2.56	5.05	10.37
Sesquiterpene Hydrocarbons	2.18	2.13	2.33	2.38	-	-	-	-	-	-	0.71
Oxygenated Sesquiterpenes	2.28	2.27	2.30	2.33	-	-	-	-	-	-	0.67
Others	2.36	1.53	1.62	1.65	0.53	0.67	0.68	-	-	-	1.45
Total	93.88	93.23	94.93	96.73	96.66	94.05	95.31	93.69	96.09	95.39	93.99

* Sample obtained by combination of equivolumetric quantity of **C1**, **C2**, **C3**, **C4**, **C5**, **C6**, and **C7** EOs.

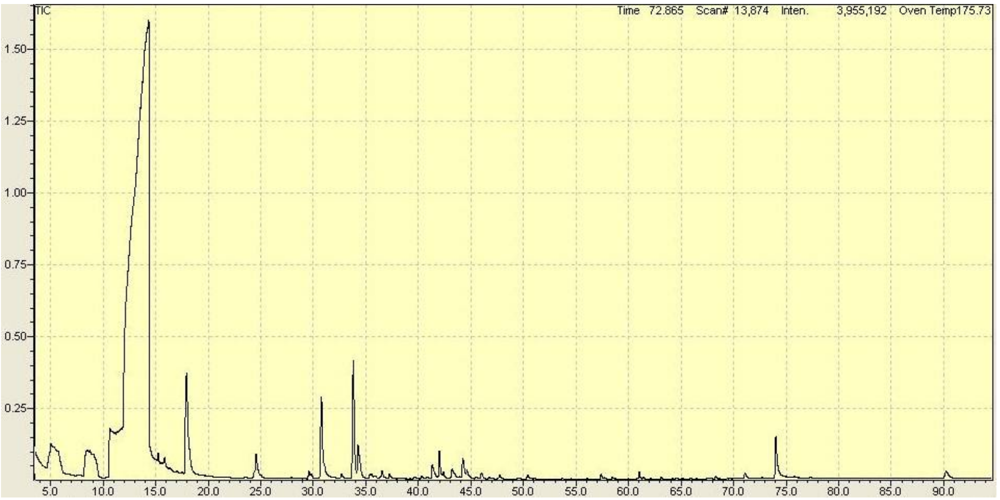

Figure S2. Chromatogram obtained by injection of *C. aurantium* 'Canaliculata' **(C1)** EO.

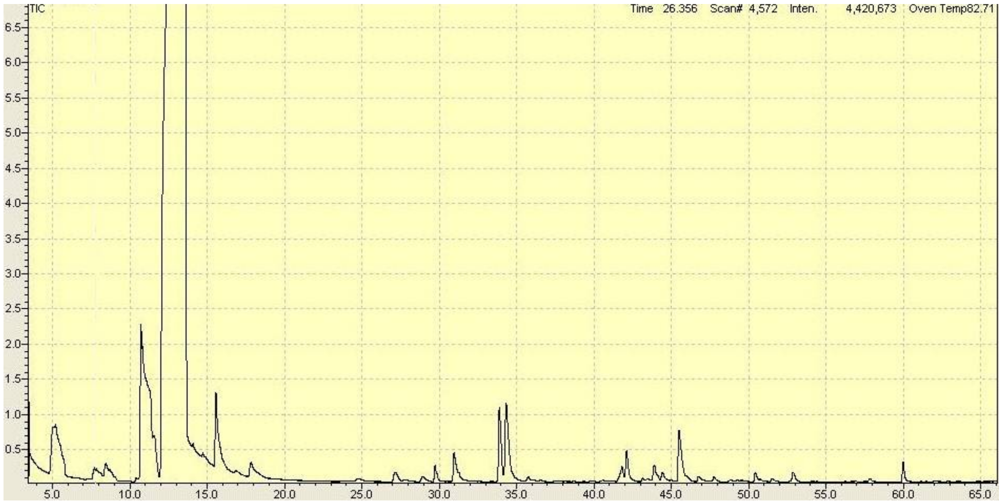

Figure S3. Chromatogram obtained by injection of *C. aurantium* 'Consolei' **(C2)** EO.

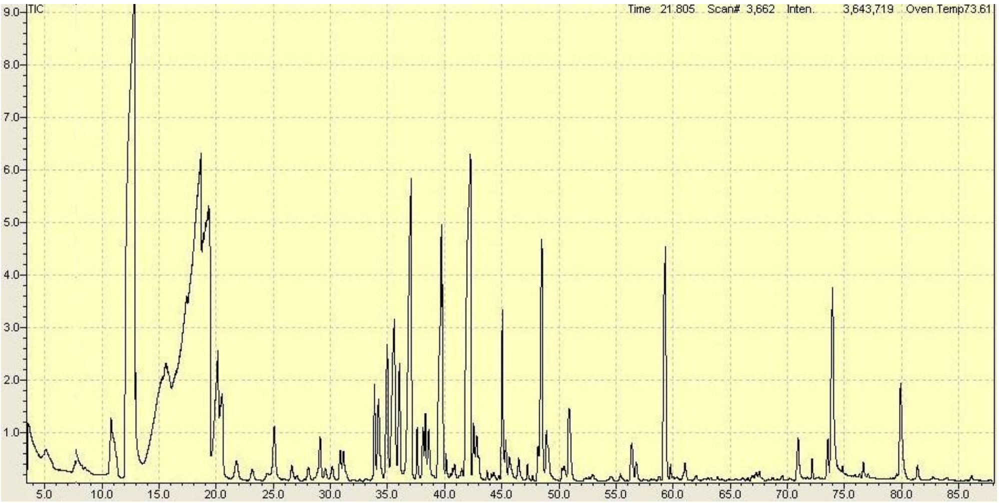

Figure S4. Chromatogram obtained by injection of *C. aurantium* 'Crispifolia' **(C3)** EO.

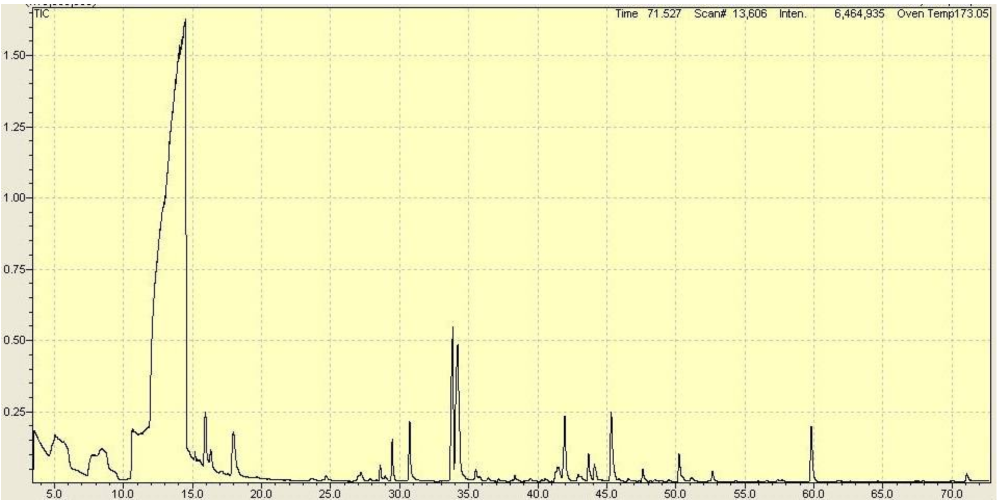

Figure S5. Chromatogram obtained by injection of *C. aurantium* 'Fasciata' **(C4)** EO.

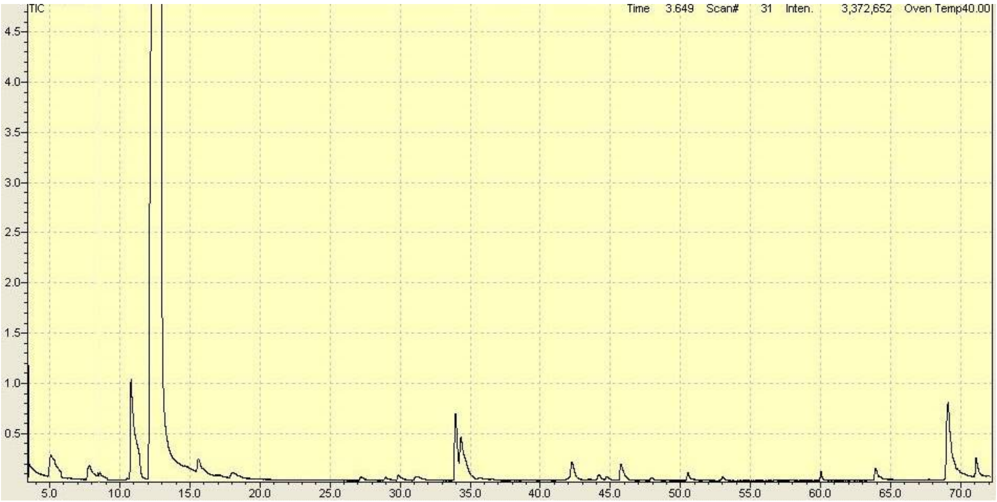

Figure S6. Chromatogram obtained by injection of *C. aurantium* 'Foetifera' **(C5)** EO.

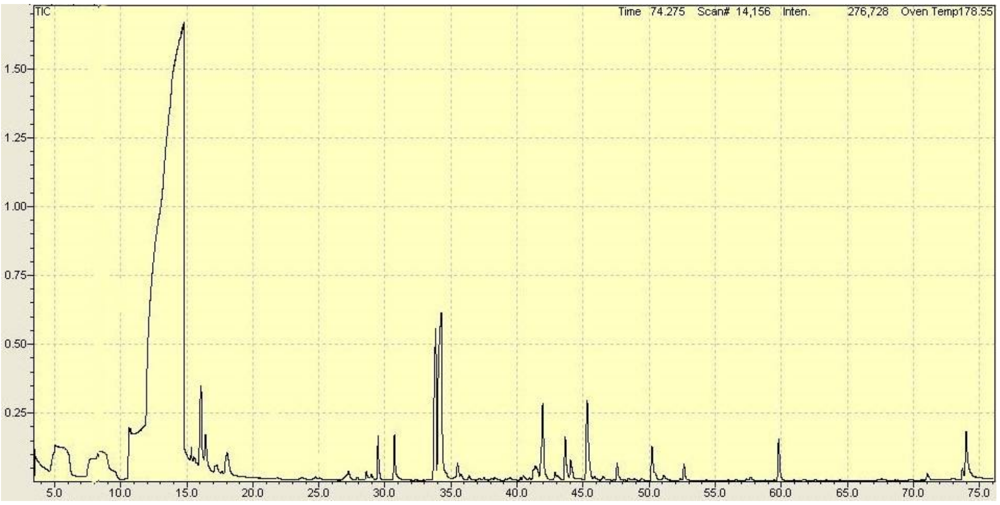

Figure S7. Chromatogram obtained by injection of *C. aurantium* 'Listata' **(C6)** EO.

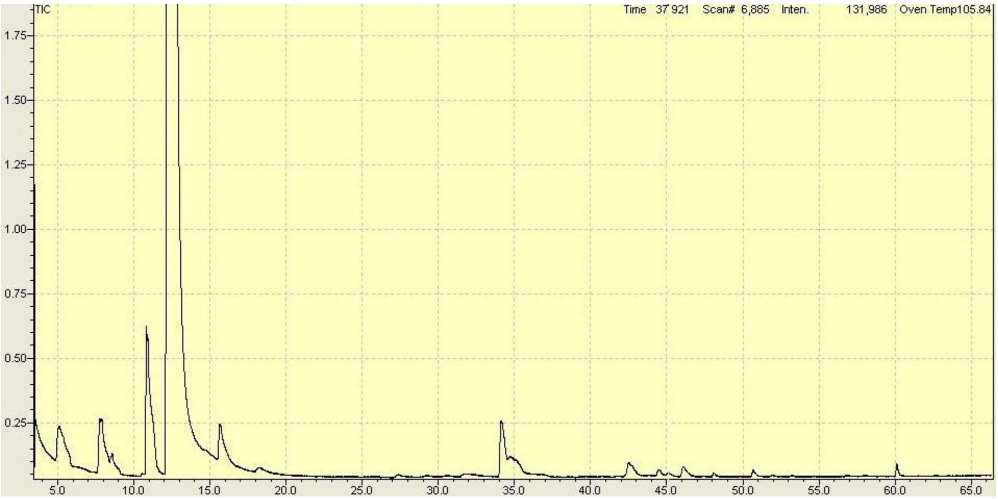

Figure S8. Chromatogram obtained by injection of *C. aurantium* 'Bizzaria' **(C7)** EO.
